# Supplementary material for: Targeted Therapy of HPV Positive and Negative Tonsillar Squamous Cell Carcinoma Cell Lines Reveals Synergy between CDK4/6, PI3K and Sometimes FGFR Inhibitors, but Rarely between PARP and WEE1 Inhibitors
Source: Viruses. 2022 Jun 23;14(7):1372. doi: 10.3390/v14071372 (PMC9320646; doi:10.3390/v14071372)
Supplement: Supplementary file 1 [file viruses-14-01372-s001.zip › viruses-1771596-supplementary.pdf]

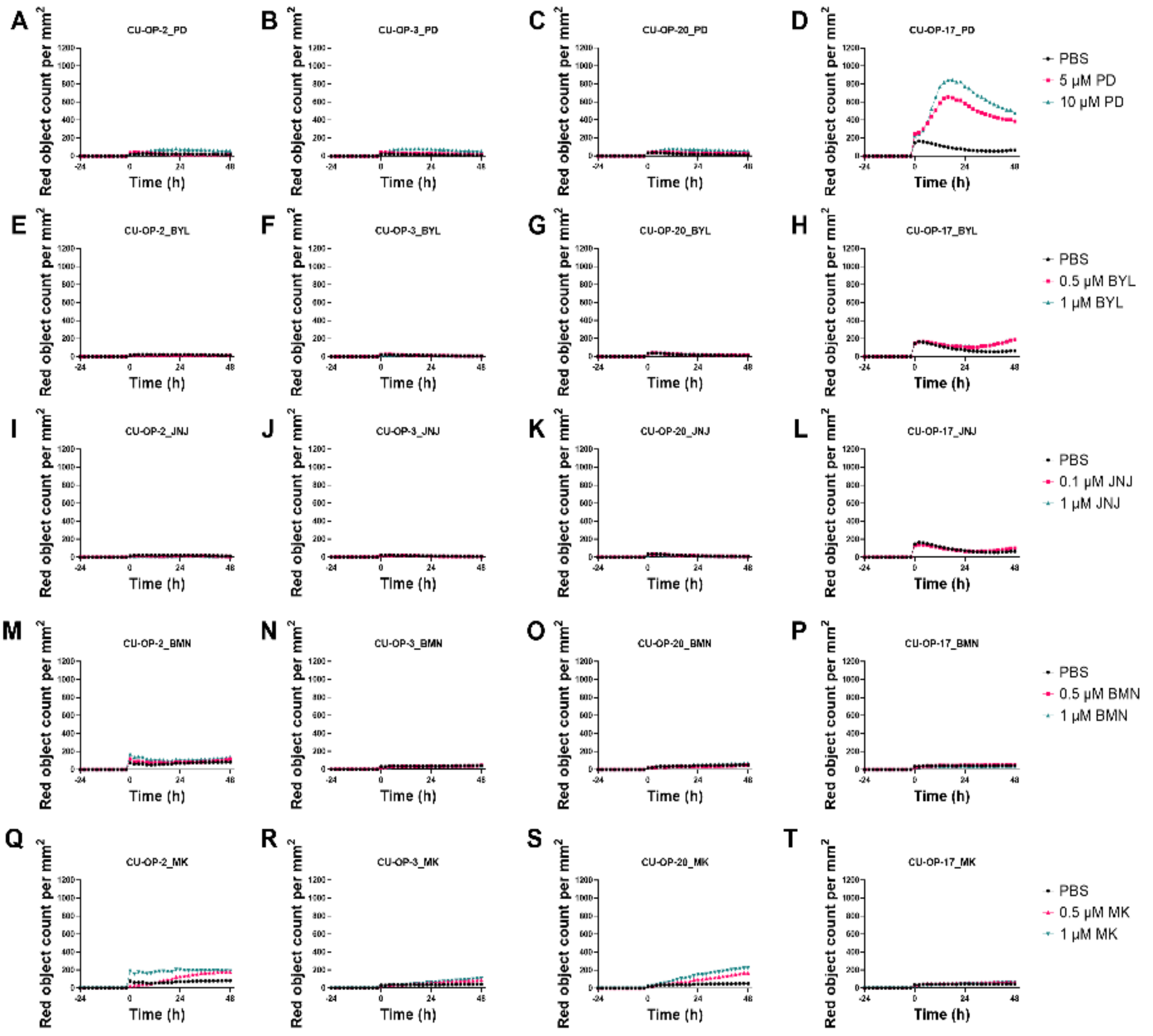

Supplementary Figure S1. Cytotoxicity of HPV^{+} CU-OP-2, CU-OP-3, CU-OP-20, and HPV^{-} CU-OP-17 cell lines upon treatment with PD-0332991, BYL719, JNJ-42756493, BMN-673 and MK-1775. Cytotoxicity of HPV^{+} CU-OP-2, CU-OP-3, CU-OP-20, and HPV^{-} CU-OP-17 after treatment with CDK4/6 inhibitor PD-0332991 (A-D), PI3K inhibitor BYL719 (E-H), FGFR inhibitor JNJ-42756493 (I-L), PARP inhibitor BMN673 (M-P) and WEE1 inhibitor MK-1775 (Q-T). The graphs represent one representative experimental run per cell line. Red objects count per mm2 denotes cytotoxicity; PD denotes PD-033299; BYL denotes BYL719; JNJ denotes JNJ-4275649; BMN denotes BMN-673 and MK denotes MK-1775.

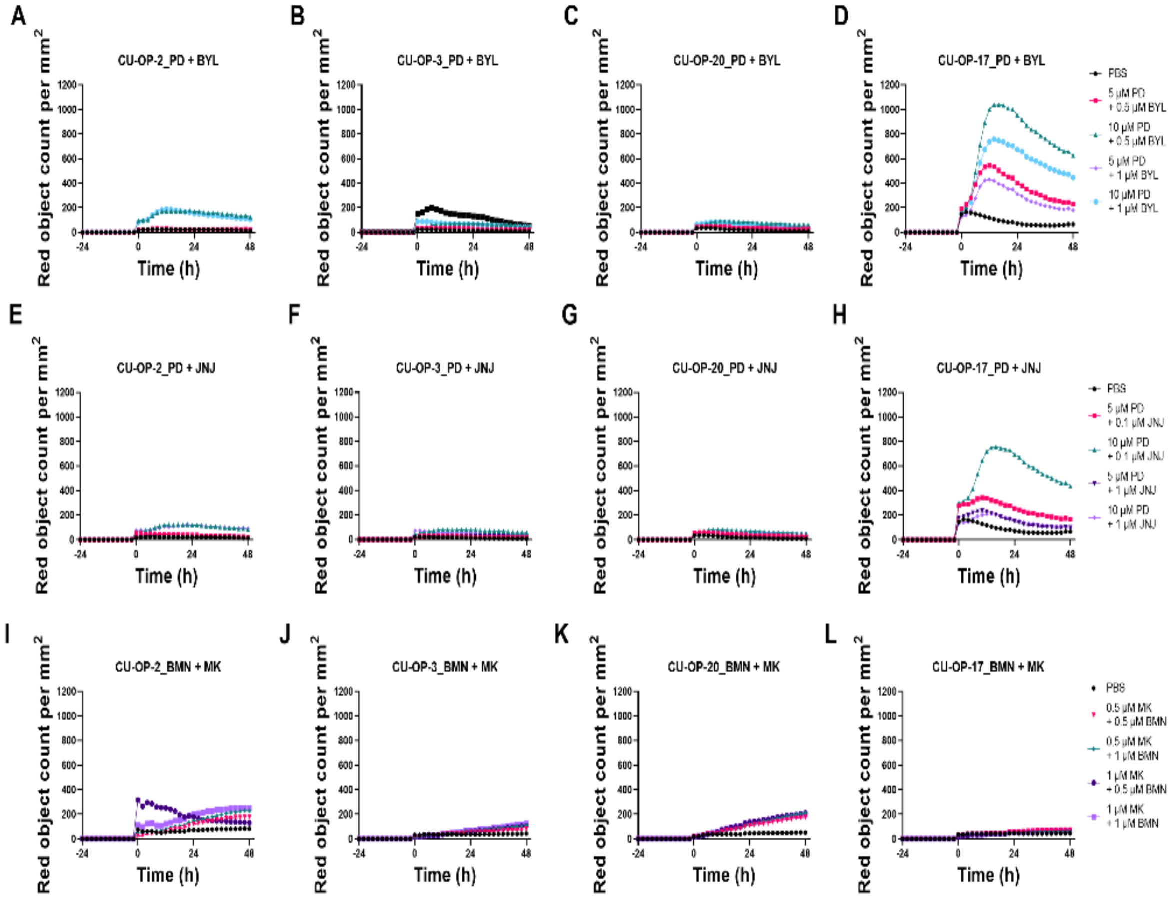

Supplementary Figure S2. Cytotoxicity of HPV+ CU-OP-2, CU-OP-3, CU-OP-20, and HPV- CU-OP-17 cell lines upon combined treatment with PD-0332991 and BYL719 or JNJ-42756493 and BMN-673 and MK-1775. Cytotoxicity of HPV+ CU-OP-2, CU-OP-3, CU-OP-20, and HPV- CU-OP-17 after treatment with CDK4/6 inhibitor PD-0332991 and PI3K inhibitor BYL719 (A-D) or with FGFR inhibitor JNJ-42756493 (E-H), or with the PARP inhibitor BMN673 and WEE1 inhibitor MK-1775 (I-L). The graphs represent one experimental run per cell line. Red objects count per mm2 denotes cytotoxicity; PD denotes PD-0332991; BYL denotes BYL719; JNJ denotes JNJ-42756493;BMN denotes BMN-673 and MK denotes MK-1775.
